# Crystal structure of bis­(μ_2_-5-nona­noylquinolin-8-olato)bis­[aqua­dichlorido­indium(III)]

**DOI:** 10.1107/S205698902400882X

**Published:** 2024-09-17

**Authors:** Betty Fuhrmann, Eric Meier, Monika Mazik

**Affiliations:** a Technische Universität Bergakademie Freiberg, Leipziger Str. 29, D-09596 Freiberg/Sachsen, Germany; Tokyo University of Science, Japan

**Keywords:** crystal structure, indium complex, quinoline, 8-hy­droxy­quinoline, hydrogen bonds, van der Waals interactions

## Abstract

Crystallization of 5-nona­noyl-8-hy­droxy­quinoline in the presence of InCl_3_ in aceto­nitrile yields a dinuclear In^III^ complex crystallizing in the space group *P*. In this complex, [In_2_(C_18_H_22_NO_2_)_2_Cl_4_(H_2_O)_2_], each indium ion is sixfold coordinated by two chloride ions, one water mol­ecule and two 8-quinolino­late ions. The crystal of the title complex is composed of two-dimensional supra­molecular aggregates, resulting from the linkage of the O_water_—H⋯O=C and O_water_—H⋯Cl hydrogen bonds as well as bifurcated C_arene_—H⋯Cl contacts.

## Abstract 2

An analysis of the complex structure obtained by crystallization of 5-nona­noyl-8-hy­droxy­quinoline and InCl_3_ in aceto­nitrile is reported.

## 1. Chemical context

As a result of the remarkable complexing properties of 8-hy­droxy­quinoline and its substituted derivatives towards various metal ions, their use as extracting agents for these ionic substrates has received much attention (for examples, see: Uhlemann *et al.*, 1984 ▸; Filik *et al.*, 1994 ▸; Gloe *et al.*, 1996 ▸; Yamada *et al.*, 2006 ▸). In addition, their application in the formation of luminescent coordination compounds has been the subject of intensive research (Matsumura *et al.*, 1996 ▸; Montes *et al.*, 2006 ▸; Feng *et al.*, 2007 ▸, 2008 ▸). Furthermore, 8-hy­droxy­quinoline-based building blocks have been used to construct artificial receptors, such as carbohydrate receptors (Mazik *et al.*, 2011 ▸; Geffert *et al.*, 2013 ▸), and have formed the basis for the development of various supra­molecular architectures (Albrecht *et al.*, 2008 ▸).

Our previous studies on the extraction of indium ions by 8-hy­droxy­quinolines bearing alkanoyl or alkyl groups of different chain lengths showed that the 5-alkanoyl derivatives are more effective indium extractors than the analogues containing 5-alkyl-, 7-alkanoyl- or 7-alkyl substituents (Schulze *et al.*, 2019 ▸). The derivative with the *n*-nona­noyl group at the 5-position proved to be a particularly effective extractor for indium ions, showing not only the best selectivity for indium over iron and zinc ions, but also the most favorable extraction kinetics under the chosen experimental conditions.
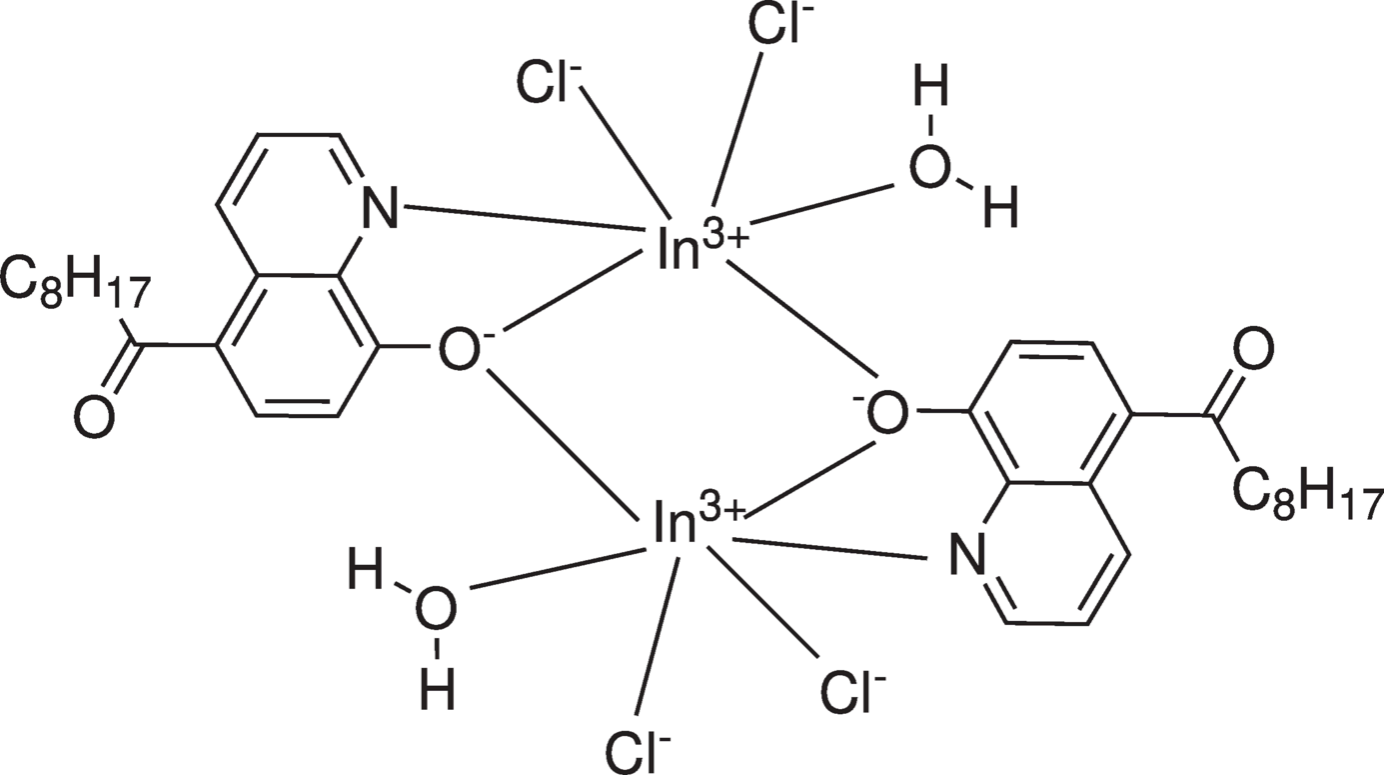


In this article we describe the crystal structure of a dinuclear In^III^ complex obtained by crystallization of 5-nona­noyl-8-hy­droxy­quinoline in the presence of InCl_3_ in aceto­nitrile.

## 2. Structural commentary

**Figure 1:**
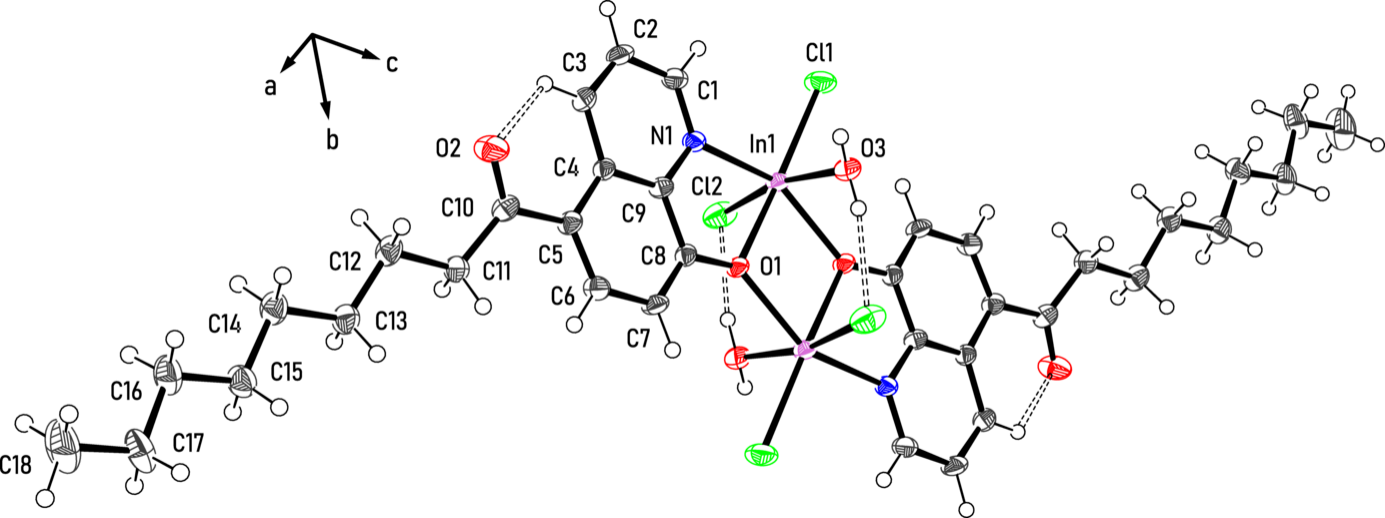
Perspective view of the mol­ecular structure of the title complex including the labeling of atoms in the asymmetric unit. The ellipsoids correspond to the thermal displacement at 50% probability.

The title complex crystallizes in the centrosymmetric space group *P*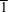 with one half of the complex in the asymmetric unit of the cell. This structural motif is expanded by an inversion center to form a dinuclear complex as depicted in Fig. 1 ▸. Within the asymmetric unit, the indium ion is fivefold coordinated *via* one water mol­ecule and two chloride ions as well as the atoms N1 and O1 of the bidentate quinolino­late ligand. The sixth coordination site of the metal center is occupied by the quinolino­late oxygen atom O1 of the inversion-related fragment of the complex, so that each In^III^ center adopts a distorted octa­hedral coordination geometry of the composition NO_3_Cl_2_. The In—*Y* bond lengths (*Y* = N, O, Cl) are listed in Table 1 ▸ and range between 2.17 and 2.43 Å. The nona­noyl fragment of the quinolino­late ligand exists in an elongated conformation. The complex structure is stabilized by intra­molecular hydrogen bonds involving the water hydrogen atom H3*B* and the chloride ion Cl2 [*d*(H⋯Cl) = 2.27 (4) Å, O—H⋯Cl = 165 (4)°] as well as C—H⋯O contacts between the nona­noyl oxygen atom O2 and the arene hydrogen atom H3 [*d*(H⋯O) = 2.21 Å, C—H⋯O = 122°].

## 3. Supra­molecular features

**Figure 2:**
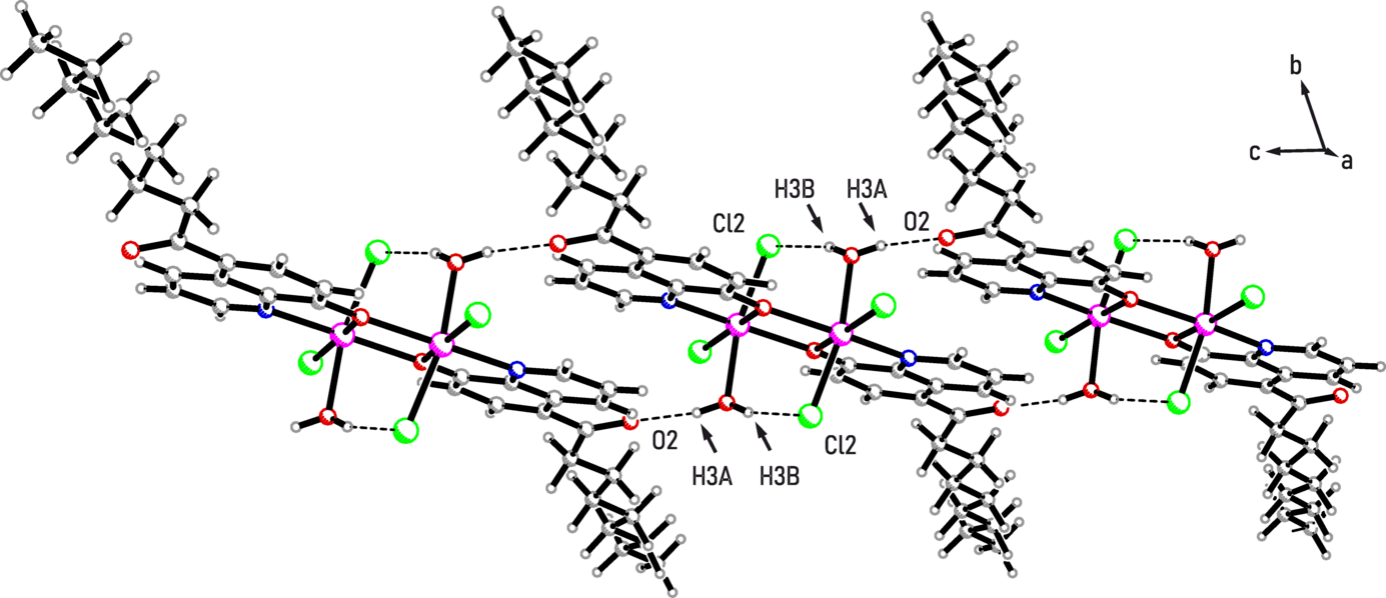
Supra­molecular chain formed by strong hydrogen bonds; color code: N – blue, O – red, Cl – green, In – magenta, C/H – gray.

**Figure 3:**
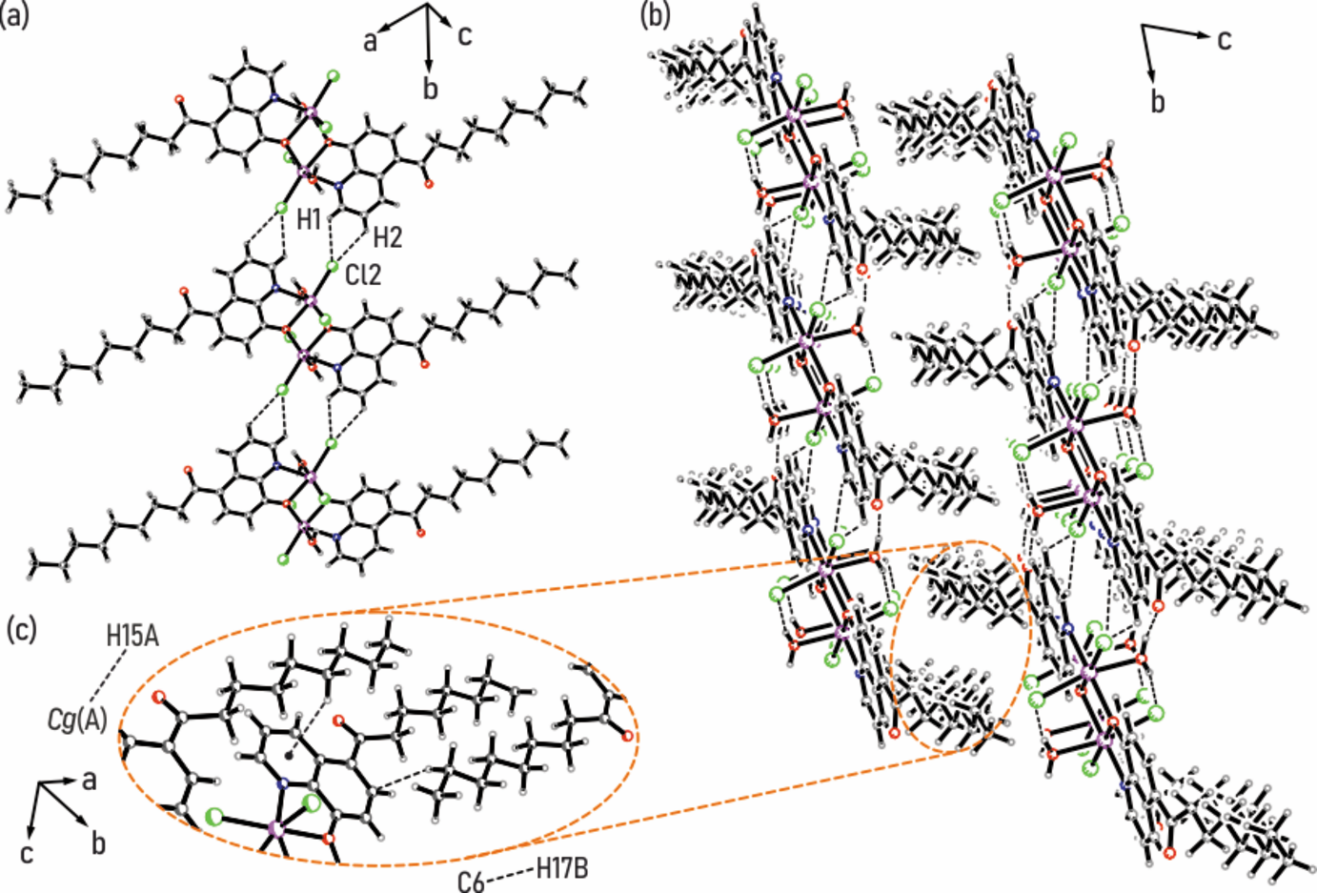
(*a*) Chain-like association of complex mol­ecules *via* C—H⋯Cl inter­actions; color code: N – blue, O – red, Cl – green, In – magenta, C/H – gray. (*b*) Excerpt of the packing structure showing two supra­molecular networks assembled *via* hydrogen bonds (dashed lines). Their mutual inter­actions are largely restricted to dispersive forces between inter­locking aliphatic moieties. (*c*) Graphical representation of weak inter­actions in which the aliphatic substituents participate.

Regarding the packing behavior of the dinuclear complexes, hydrogen bonds play an important role. On one hand, the observed inter­action between the water hydrogen atom H3*A* and the carbonyl oxygen atom O2 [*d* =1.84 (4) Å, C—H⋯O = 168 (5)°; see Fig. 2 ▸] of adjacent mol­ecules leads to the formation of infinite supra­molecular chains in the [10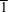] direction. On the other hand, weaker C_arene_—H⋯Cl hydrogen bonds with the chloride ion Cl2 acting as a bifurcated acceptor for H1 and H2 of the neighboring mol­ecule (see Fig. 3 ▸*a* and Table 1 ▸) crosslink these chains along the *b* axis to form a two-dimensional supra­molecular network.

The packing structure of the complex shown in Fig. 3 ▸*b* indicates that the parallel orientation of the aliphatic C_8_H_17_ units has a strong influence on the cohesion of the crystal structure by van der Waals forces. They are supported by C—H⋯π inter­actions between H15*A* and the heterocyclic subunit (*A*) of the quinoline scaffold (see Fig. 3 ▸*c* and Table 1 ▸). In addition, the inter­actions between H17*B* and C6 of the quinoline ring appear to have a stabilizing effect. Other contacts involving the aromatic rings are absent in the crystal, as the closest *Cg*⋯*Cg* distances between their centroids amount to about 4.2 Å.

## 4. Database survey

A search in the Cambridge Structural Database (CSD, Version 5.44, update of September 2023; Groom *et al.*, 2016 ▸) for indium complexes with ligands based on 8-hy­droxy­quinoline yielded 15 hits. The quinolines often occur as individual ligands within the complexes, but sometimes they are also incorporated as a subunit of larger mol­ecules.

Common to all complexes is that the 8-quinolino­late acts as a chelating ligand, complexing the indium ion *via* its oxygen and ring nitro­gen atom. The reported indium complexes are predominantly mononuclear. However, three dinuclear complexes are also included in the database entries mentioned above. In the case of the dinuclear chelate complexes, the indium ions possess coordination numbers of six (ALESES; Alexander *et al.*, 2021 ▸) or five (SOMYOL, SOMZEC; Kwak *et al.*, 2019 ▸). The mononuclear complexes mostly have a coordination number of six, but occasionally the indium ion is coordinated five-, seven- or eightfold.

In the crystal structure with the reference code ALESES, the indium ion adopts a coordination environment of the composition N_2_O_3_Cl. Since this complex lacks a quinoline-bound keto group and no water mol­ecule is involved, a strand-like association as in the crystal structure of the title complex cannot be observed. Instead, weak C_ar­yl_—H⋯Cl and C_ar­yl_—H⋯O hydrogen bonds as well as π⋯π contacts between the quinoline units of adjacent complexes lead to the formation of two-dimensional supra­molecular networks. The packing structures of the complexes with the reference codes SOMYOL and SOMZEC, containing NO_2_C_2_-coordinated indium ions, consist of an infinite strand-like arrangement of mol­ecules connected by π⋯π inter­actions similar to those mentioned above.

## 5. Synthesis and crystallization

5-Nonanoyl-8-hy­droxy­quinoline (50 mg, 0.18 mmol) and indium(III) chloride (116 mg, 0.52 mmol) were stirred in methanol (5 mL) for 30 min at room temperature and the solvent was removed under vacuum. Afterwards the residue was crystallized by slow evaporation from aceto­nitrile. 5-Nonanoyl-8-hy­droxy­quinoline was synthesized according to the literature procedure (Uhlemann *et al.*, 1981 ▸).

## 6. Refinement

Crystal data, data collection and structure refinement details are summarized in Table 2 ▸. All non-hydrogen atoms were refined anisotropically. Both hydrogen atoms of the water mol­ecule (H3*A*, H3*B*) were located in difference-Fourier maps and placed accordingly. The remaining hydrogen atoms were positioned geometrically and refined isotropically using a riding model, with C—H bond distances of 0.95 Å (ar­yl), 0.98 Å (methyl­ene) and 0.99 Å (meth­yl). The thermal displace­ment ellipsoids of all hydrogen atoms were set to *U*_iso_(H) = 1.2*U*_eq_(C) and *U*_iso_(H) = 1.5 *U*_eq_(C/O), the latter applying to methyl and water moieties.

The crystal was refined as a two-component non-merohedral twin, whereby the main domain makes up 72% of the crystal. The two domains were identified and integrated simultaneously *via* the *Recipe/Index/Refine* and *Integrate* modules, respectively, of the *X-AREA* program suite, followed by absorption correction and scaling of the resulting HKLF5 dataset *via* the modules *X-RED32* and *LANA*, respectively (Stoe & Cie, 2002 ▸). The reflection file employed in the subsequent refinement contained reflections from the two individual domains as well as reflections to which both domains contributed.

By recognizing twinning, the *R*-values as well as the maximum residual electron density (Table 2 ▸) improved drastically compared to the model based on untreated HKLF4 data (*R*_1_ = 7.24%, w*R*_2_ = 22.71%, maximum electron density = 3.94 e Å^−3^).

## Supplementary Material

Crystal structure: contains datablock(s) I. DOI: 10.1107/S205698902400882X/jp2009sup1.cif

Structure factors: contains datablock(s) I. DOI: 10.1107/S205698902400882X/jp2009Isup2.hkl

Supporting information file. DOI: 10.1107/S205698902400882X/jp2009Isup3.cdx

CCDC reference: 2382976

Additional supporting information:  crystallographic information; 3D view; checkCIF report

## Figures and Tables

**Table 1:** Geometric data (Å, °) for short intra- and inter­molecular inter­actions *CgA* is the centroid of the N1/C1–C4/C9 ring.

In—*Y*		In–*Y*		
In1—O1^i^	2.166 (3)	In1—N1^ii^	2.241 (4)	
In1—O1^ii^	2.209 (3)	In1—Cl1^ii^	2.382 (2)	
In1—O3^ii^	2.227 (4)	In1—Cl2^ii^	2.430 (2)	
				
*D*—H⋯*A*/*Cg*	*D*—H	H⋯*A*/*Cg*	*D*—*A*/*Cg*	*D*—H⋯*A*/*Cg*
O3—H3*A*⋯O2^iii^	0.84 (4)	1.84 (4)	2.668 (4)	168 (5)
O3—H3*B*⋯Cl2^i^	0.92 (4)	2.27 (4)	3.165 (4)	165 (4)
C1—H1⋯Cl1^iv^	0.95	2.76	3.417 (6)	127
C2—H2⋯Cl1^iv^	0.95	2.89	3.469 (5)	120
C3—H3⋯O2^ii^	0.95	2.21	2.835 (6)	122
C15—H15*A*⋯*CgA*^v^	0.99	2.94	3.766 (6)	141

Symmetry codes: (i) *x* − 1, −*y* + 1, −*z* + 1; (ii) *x*, *y*, *z*; (iii) −*x* + 2, −*y*, −*z* + 1; (iv) −*x* + 1, −*y*, −*z* + 1; (v) *x* + 1, *y*, *z*.

**Table 2:** Experimental details

Crystal data	
Chemical formula	[In_2_(C_18_H_22_NO_2_)_2_Cl_4_(H_2_O)_2_]
*M*_r_	976.20
Crystal system, space group	Triclinic, *P* 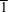
Temperature (K)	163
*a*, *b*, *c* (Å)	10.297 (4), 10.940 (4), 11.711 (5)
α, β, γ (°)	63.57 (3), 72.47 (3), 62.92 (3)
*V* (Å^3^)	1043.8 (8)
*Z*	1
Radiation type	Mo *K*α
μ (mm^−1^)	1.40
Crystal size (mm)	0.19 × 0.10 × 0.07

Data collection	
Diffractometer	Stoe Stadivari
Absorption correction	Multi-scan (*X-RED32*; Stoe & Cie, 2002 ▸)
*T*_min_, *T*_max_	0.766, 0.907
No. of measured, independent and observed [*I* > 2σ(*I*)] reflections	62899, 62899, 42801
*R*_int_	0.039

Refinement	
*R*[*F*^2^ > 2σ(*F*^2^)], *wR*(*F*^2^), *S*	0.039, 0.061, 0.88
No. of reflections	62899
No. of parameters	234
H-atom treatment	H atoms treated by a mixture of independent and constrained refinement
Δρ_max_, Δρ_min_ (e Å^−3^)	0.94, −0.74

Computer programs: *X-AREA*, *X-RED32* and *LANA* (Stoe & Cie, 2002 ▸), *SHELXT2018/2* (Sheldrick, 2015*a* ▸), *SHELXL2018/3* (Sheldrick, 2015*b* ▸), *OLEX2* (Dolomanov *et al.*, 2009 ▸), *XP* in *SHELXTL* (Sheldrick, 2008 ▸) and *ORTEP-3 for Windows* (Farrugia, 2012 ▸).

## supplementary crystallographic information

### Bis(µ2-5-nonanoylquinolin-8-olato)bis[aquadichloridoindium(III)] Crystal data

**Table:**

[In_2_(C_18_H_22_NO_2_)_2_Cl_4_(H_2_O)_2_]	*Z* = 1
*M**_r_* = 976.20	*F*(000) = 492
Triclinic, *P*1	*D*_x_ = 1.553 Mg m^−^^3^
*a* = 10.297 (4) Å	Mo *K*α radiation, λ = 0.71073 Å
*b* = 10.940 (4) Å	Cell parameters from 24127 reflections
*c* = 11.711 (5) Å	θ = 2.2–28.7°
α = 63.57 (3)°	µ = 1.40 mm^−^^1^
β = 72.47 (3)°	*T* = 163 K
γ = 62.92 (3)°	Plate, colourless
*V* = 1043.8 (8) Å^3^	0.19 × 0.10 × 0.07 mm

### Bis(µ2-5-nonanoylquinolin-8-olato)bis[aquadichloridoindium(III)] Data collection

**Table:**

Stoe Stadivari diffractometer	62899 independent reflections
Radiation source: Primux 50 Mo	42801 reflections with *I* > 2σ(*I*)
Graded multilayer mirror monochromator	*R*_int_ = 0.039
Detector resolution: 5.81 pixels mm^-1^	θ_max_ = 27.5°, θ_min_ = 2.2°
rotation method, ω scans	*h* = −13→13
Absorption correction: multi-scan (X-Red32; Stoe & Cie, 2002)	*k* = −14→14
*T*_min_ = 0.766, *T*_max_ = 0.907	*l* = −15→15
62899 measured reflections	

### Bis(µ2-5-nonanoylquinolin-8-olato)bis[aquadichloridoindium(III)] Refinement

**Table:**

Refinement on *F*^2^	Primary atom site location: dual
Least-squares matrix: full	Hydrogen site location: mixed
*R*[*F*^2^ > 2σ(*F*^2^)] = 0.039	H atoms treated by a mixture of independent and constrained refinement
*wR*(*F*^2^) = 0.061	*w* = 1/[σ^2^(*F*_o_^2^) + (0.0118*P*)^2^] where *P* = (*F*_o_^2^ + 2*F*_c_^2^)/3
*S* = 0.88	(Δ/σ)_max_ = 0.001
62899 reflections	Δρ_max_ = 0.94 e Å^−^^3^
234 parameters	Δρ_min_ = −0.74 e Å^−^^3^
0 restraints	

### Bis(µ2-5-nonanoylquinolin-8-olato)bis[aquadichloridoindium(III)] Special details

**Table:**

Geometry. All esds (except the esd in the dihedral angle between two l.s. planes) are estimated using the full covariance matrix. The cell esds are taken into account individually in the estimation of esds in distances, angles and torsion angles; correlations between esds in cell parameters are only used when they are defined by crystal symmetry. An approximate (isotropic) treatment of cell esds is used for estimating esds involving l.s. planes.
Refinement. Refined as a 2-component twin.

### Bis(µ2-5-nonanoylquinolin-8-olato)bis[aquadichloridoindium(III)] Fractional atomic coordinates and isotropic or equivalent isotropic displacement parameters (Å^2^)

**Table:**

	*x*	*y*	*z*	*U*_iso_*/*U*_eq_	
C1	0.7219 (4)	0.0957 (5)	0.3966 (5)	0.0270 (12)	
H1	0.6420	0.0662	0.4143	0.032*	
C2	0.8613 (4)	0.0088 (5)	0.3551 (4)	0.0284 (12)	
H2	0.8760	−0.0788	0.3459	0.034*	
C3	0.9763 (4)	0.0515 (5)	0.3279 (4)	0.0260 (12)	
H3	1.0710	−0.0055	0.2973	0.031*	
C4	0.9562 (4)	0.1795 (5)	0.3447 (4)	0.0202 (11)	
C5	1.0678 (4)	0.2360 (5)	0.3187 (4)	0.0226 (11)	
C6	1.0297 (4)	0.3609 (5)	0.3417 (5)	0.0266 (12)	
H6	1.1039	0.3973	0.3255	0.032*	
C7	0.8868 (4)	0.4380 (5)	0.3880 (4)	0.0260 (12)	
H7	0.8668	0.5223	0.4053	0.031*	
C8	0.7761 (4)	0.3912 (5)	0.4080 (4)	0.0202 (11)	
C9	0.8106 (4)	0.2605 (5)	0.3875 (4)	0.0202 (11)	
C10	1.2233 (4)	0.1595 (5)	0.2701 (5)	0.0252 (12)	
C11	1.3194 (4)	0.2486 (5)	0.2001 (5)	0.0274 (12)	
H11A	1.3243	0.2894	0.2584	0.033*	
H11B	1.2720	0.3328	0.1255	0.033*	
C12	1.4756 (4)	0.1645 (5)	0.1521 (5)	0.0301 (13)	
H12A	1.4734	0.1317	0.0866	0.036*	
H12B	1.5228	0.0760	0.2244	0.036*	
C13	1.5642 (4)	0.2633 (5)	0.0937 (5)	0.0312 (13)	
H13A	1.5621	0.2980	0.1595	0.037*	
H13B	1.5156	0.3510	0.0216	0.037*	
C14	1.7255 (4)	0.1892 (5)	0.0439 (5)	0.0327 (13)	
H14A	1.7693	0.0905	0.1089	0.039*	
H14B	1.7296	0.1761	−0.0356	0.039*	
C15	1.8144 (4)	0.2809 (5)	0.0162 (5)	0.0340 (13)	
H15A	1.8062	0.2969	0.0953	0.041*	
H15B	1.7710	0.3785	−0.0501	0.041*	
C16	1.9767 (4)	0.2114 (5)	−0.0297 (5)	0.0404 (14)	
H16A	2.0183	0.1097	0.0320	0.048*	
H16B	1.9861	0.2058	−0.1140	0.048*	
C17	2.0644 (4)	0.2983 (6)	−0.0424 (5)	0.0453 (15)	
H17A	2.0549	0.3039	0.0420	0.054*	
H17B	2.0225	0.4001	−0.1039	0.054*	
C18	2.2278 (4)	0.2295 (6)	−0.0887 (5)	0.066 (2)	
H18A	2.2719	0.1317	−0.0248	0.099*	
H18B	2.2781	0.2924	−0.1001	0.099*	
H18C	2.2379	0.2203	−0.1707	0.099*	
Cl1	0.34019 (11)	0.21319 (13)	0.53300 (14)	0.0374 (4)	
Cl2	0.38785 (12)	0.53711 (14)	0.27233 (13)	0.0403 (4)	
In1	0.47713 (3)	0.36247 (4)	0.47732 (4)	0.02240 (9)	
N1	0.6970 (3)	0.2161 (4)	0.4119 (4)	0.0220 (9)	
O1	0.6352 (2)	0.4620 (3)	0.4468 (3)	0.0220 (8)	
O2	1.2707 (3)	0.0302 (3)	0.2842 (4)	0.0403 (10)	
O3	0.5333 (3)	0.2442 (3)	0.6779 (3)	0.0288 (9)	
H3A	0.588 (4)	0.154 (5)	0.700 (5)	0.043*	
H3B	0.559 (4)	0.294 (5)	0.707 (4)	0.043*	

### Bis(µ2-5-nonanoylquinolin-8-olato)bis[aquadichloridoindium(III)] Atomic displacement parameters (Å^2^)

**Table:**

	*U*^11^	*U*^22^	*U*^33^	*U*^12^	*U*^13^	*U*^23^
C1	0.026 (3)	0.023 (3)	0.035 (4)	−0.012 (2)	0.000 (2)	−0.013 (3)
C2	0.029 (2)	0.019 (3)	0.040 (4)	−0.007 (2)	0.000 (2)	−0.017 (3)
C3	0.023 (2)	0.019 (3)	0.030 (3)	−0.003 (2)	0.001 (2)	−0.012 (3)
C4	0.021 (2)	0.016 (3)	0.021 (3)	−0.004 (2)	−0.002 (2)	−0.007 (2)
C5	0.018 (2)	0.019 (3)	0.029 (3)	−0.003 (2)	−0.003 (2)	−0.011 (2)
C6	0.016 (2)	0.024 (3)	0.039 (4)	−0.007 (2)	−0.001 (2)	−0.014 (3)
C7	0.023 (2)	0.019 (3)	0.039 (4)	−0.006 (2)	−0.001 (2)	−0.017 (3)
C8	0.019 (2)	0.016 (3)	0.021 (3)	−0.003 (2)	−0.002 (2)	−0.007 (2)
C9	0.019 (2)	0.016 (3)	0.025 (3)	−0.003 (2)	−0.006 (2)	−0.008 (2)
C10	0.021 (2)	0.024 (3)	0.030 (3)	−0.003 (2)	−0.007 (2)	−0.013 (3)
C11	0.021 (2)	0.027 (3)	0.035 (4)	−0.009 (2)	0.002 (2)	−0.015 (3)
C12	0.018 (2)	0.033 (3)	0.039 (4)	−0.004 (2)	−0.001 (2)	−0.020 (3)
C13	0.023 (2)	0.033 (3)	0.037 (4)	−0.009 (2)	0.000 (2)	−0.017 (3)
C14	0.022 (2)	0.037 (3)	0.037 (4)	−0.008 (2)	0.000 (2)	−0.017 (3)
C15	0.026 (3)	0.036 (3)	0.038 (4)	−0.013 (2)	0.000 (2)	−0.013 (3)
C16	0.026 (3)	0.056 (4)	0.040 (4)	−0.018 (3)	0.004 (2)	−0.020 (3)
C17	0.032 (3)	0.068 (4)	0.036 (4)	−0.029 (3)	0.003 (3)	−0.013 (3)
C18	0.033 (3)	0.106 (6)	0.065 (5)	−0.036 (3)	0.008 (3)	−0.035 (4)
Cl1	0.0307 (6)	0.0322 (8)	0.0596 (11)	−0.0178 (6)	0.0014 (6)	−0.0234 (8)
Cl2	0.0544 (8)	0.0296 (8)	0.0394 (10)	−0.0098 (7)	−0.0191 (7)	−0.0124 (7)
In1	0.01802 (15)	0.01785 (17)	0.0336 (2)	−0.00564 (12)	−0.00088 (14)	−0.01387 (16)
N1	0.0185 (18)	0.018 (2)	0.030 (3)	−0.0068 (17)	0.0014 (17)	−0.011 (2)
O1	0.0158 (15)	0.0197 (17)	0.032 (2)	−0.0057 (13)	0.0026 (13)	−0.0154 (17)
O2	0.0220 (17)	0.022 (2)	0.069 (3)	−0.0035 (15)	0.0001 (17)	−0.018 (2)
O3	0.0322 (18)	0.0194 (19)	0.032 (2)	−0.0070 (15)	−0.0026 (16)	−0.0109 (18)

### Bis(µ2-5-nonanoylquinolin-8-olato)bis[aquadichloridoindium(III)] Geometric parameters (Å, º)

**Table:**

C1—N1	1.308 (5)	C13—H13A	0.9900
C1—C2	1.396 (5)	C13—H13B	0.9900
C1—H1	0.9500	C14—C15	1.522 (5)
C2—C3	1.367 (5)	C14—H14A	0.9900
C2—H2	0.9500	C14—H14B	0.9900
C3—C4	1.414 (5)	C15—C16	1.524 (5)
C3—H3	0.9500	C15—H15A	0.9900
C4—C9	1.420 (5)	C15—H15B	0.9900
C4—C5	1.437 (5)	C16—C17	1.522 (6)
C5—C6	1.368 (6)	C16—H16A	0.9900
C5—C10	1.497 (5)	C16—H16B	0.9900
C6—C7	1.402 (5)	C17—C18	1.534 (5)
C6—H6	0.9500	C17—H17A	0.9900
C7—C8	1.372 (5)	C17—H17B	0.9900
C7—H7	0.9500	C18—H18A	0.9800
C8—O1	1.344 (4)	C18—H18B	0.9800
C8—C9	1.421 (6)	C18—H18C	0.9800
C9—N1	1.370 (5)	Cl1—In1	2.3825 (14)
C10—O2	1.215 (5)	Cl2—In1	2.4302 (19)
C10—C11	1.511 (5)	In1—O1^i^	2.166 (3)
C11—C12	1.522 (5)	In1—O1	2.209 (3)
C11—H11A	0.9900	In1—O3	2.227 (4)
C11—H11B	0.9900	In1—N1	2.241 (3)
C12—C13	1.528 (5)	O1—In1^i^	2.166 (3)
C12—H12A	0.9900	O3—H3A	0.84 (4)
C12—H12B	0.9900	O3—H3B	0.92 (4)
C13—C14	1.538 (5)		
			
N1—C1—C2	122.5 (4)	C15—C14—H14B	109.4
N1—C1—H1	118.7	C13—C14—H14B	109.4
C2—C1—H1	118.7	H14A—C14—H14B	108.0
C3—C2—C1	119.0 (4)	C14—C15—C16	114.1 (4)
C3—C2—H2	120.5	C14—C15—H15A	108.7
C1—C2—H2	120.5	C16—C15—H15A	108.7
C2—C3—C4	120.8 (4)	C14—C15—H15B	108.7
C2—C3—H3	119.6	C16—C15—H15B	108.7
C4—C3—H3	119.6	H15A—C15—H15B	107.6
C3—C4—C9	116.0 (4)	C17—C16—C15	112.3 (4)
C3—C4—C5	125.9 (4)	C17—C16—H16A	109.1
C9—C4—C5	118.0 (4)	C15—C16—H16A	109.1
C6—C5—C4	118.3 (4)	C17—C16—H16B	109.1
C6—C5—C10	120.2 (4)	C15—C16—H16B	109.1
C4—C5—C10	121.4 (4)	H16A—C16—H16B	107.9
C5—C6—C7	123.5 (4)	C16—C17—C18	112.7 (4)
C5—C6—H6	118.2	C16—C17—H17A	109.0
C7—C6—H6	118.2	C18—C17—H17A	109.0
C8—C7—C6	119.7 (4)	C16—C17—H17B	109.0
C8—C7—H7	120.2	C18—C17—H17B	109.0
C6—C7—H7	120.2	H17A—C17—H17B	107.8
O1—C8—C7	123.6 (4)	C17—C18—H18A	109.5
O1—C8—C9	117.5 (3)	C17—C18—H18B	109.5
C7—C8—C9	118.9 (4)	H18A—C18—H18B	109.5
N1—C9—C4	121.8 (4)	C17—C18—H18C	109.5
N1—C9—C8	116.8 (4)	H18A—C18—H18C	109.5
C4—C9—C8	121.4 (4)	H18B—C18—H18C	109.5
O2—C10—C5	121.4 (4)	O1^i^—In1—O1	72.65 (11)
O2—C10—C11	120.6 (4)	O1^i^—In1—O3	78.60 (12)
C5—C10—C11	118.0 (4)	O1—In1—O3	84.69 (12)
C10—C11—C12	115.3 (4)	O1^i^—In1—N1	144.79 (11)
C10—C11—H11A	108.5	O1—In1—N1	73.40 (11)
C12—C11—H11A	108.5	O3—In1—N1	89.29 (13)
C10—C11—H11B	108.5	O1^i^—In1—Cl1	112.71 (8)
C12—C11—H11B	108.5	O1—In1—Cl1	169.15 (8)
H11A—C11—H11B	107.5	O3—In1—Cl1	87.16 (9)
C11—C12—C13	109.9 (3)	N1—In1—Cl1	99.38 (10)
C11—C12—H12A	109.7	O1^i^—In1—Cl2	89.09 (9)
C13—C12—H12A	109.7	O1—In1—Cl2	91.53 (9)
C11—C12—H12B	109.7	O3—In1—Cl2	167.69 (9)
C13—C12—H12B	109.7	N1—In1—Cl2	100.88 (11)
H12A—C12—H12B	108.2	Cl1—In1—Cl2	97.88 (6)
C12—C13—C14	114.7 (4)	C1—N1—C9	119.8 (3)
C12—C13—H13A	108.6	C1—N1—In1	124.9 (3)
C14—C13—H13A	108.6	C9—N1—In1	115.3 (3)
C12—C13—H13B	108.6	C8—O1—In1^i^	134.6 (2)
C14—C13—H13B	108.6	C8—O1—In1	117.0 (2)
H13A—C13—H13B	107.6	In1^i^—O1—In1	107.35 (11)
C15—C14—C13	111.3 (4)	In1—O3—H3A	118 (3)
C15—C14—H14A	109.4	In1—O3—H3B	115 (3)
C13—C14—H14A	109.4	H3A—O3—H3B	112 (4)
			
N1—C1—C2—C3	−0.8 (7)	C4—C5—C10—O2	−20.8 (7)
C1—C2—C3—C4	1.7 (7)	C6—C5—C10—C11	−23.3 (6)
C2—C3—C4—C9	−1.6 (7)	C4—C5—C10—C11	157.9 (4)
C2—C3—C4—C5	−179.6 (4)	O2—C10—C11—C12	−0.7 (7)
C3—C4—C5—C6	−179.3 (5)	C5—C10—C11—C12	−179.4 (4)
C9—C4—C5—C6	2.8 (7)	C10—C11—C12—C13	−175.2 (4)
C3—C4—C5—C10	−0.5 (7)	C11—C12—C13—C14	178.9 (4)
C9—C4—C5—C10	−178.4 (4)	C12—C13—C14—C15	−166.9 (4)
C4—C5—C6—C7	−0.8 (7)	C13—C14—C15—C16	178.2 (4)
C10—C5—C6—C7	−179.6 (4)	C14—C15—C16—C17	−173.9 (4)
C5—C6—C7—C8	−2.3 (7)	C15—C16—C17—C18	−179.9 (4)
C6—C7—C8—O1	−177.0 (4)	C2—C1—N1—C9	−0.3 (7)
C6—C7—C8—C9	3.2 (7)	C2—C1—N1—In1	179.8 (3)
C3—C4—C9—N1	0.5 (6)	C4—C9—N1—C1	0.5 (7)
C5—C4—C9—N1	178.6 (4)	C8—C9—N1—C1	−179.1 (4)
C3—C4—C9—C8	180.0 (4)	C4—C9—N1—In1	−179.6 (3)
C5—C4—C9—C8	−1.8 (7)	C8—C9—N1—In1	0.8 (5)
O1—C8—C9—N1	−1.4 (6)	C7—C8—O1—In1^i^	−12.1 (7)
C7—C8—C9—N1	178.4 (4)	C9—C8—O1—In1^i^	167.7 (3)
O1—C8—C9—C4	179.1 (4)	C7—C8—O1—In1	−178.5 (4)
C7—C8—C9—C4	−1.1 (7)	C9—C8—O1—In1	1.3 (5)
C6—C5—C10—O2	157.9 (5)		

Symmetry code: (i) −*x*+1, −*y*+1, −*z*+1.

### Bis(µ2-5-nonanoylquinolin-8-olato)bis[aquadichloridoindium(III)] Hydrogen-bond geometry (Å, º)

**Table:**

*D*—H···*A*	*D*—H	H···*A*	*D*···*A*	*D*—H···*A*
O3—H3*A*···O2^ii^	0.84 (4)	1.84 (4)	2.668 (4)	168 (5)
O3—H3*B*···Cl2^i^	0.92 (4)	2.27 (4)	3.165 (4)	165 (4)

Symmetry codes: (i) −*x*+1, −*y*+1, −*z*+1; (ii) −*x*+2, −*y*, −*z*+1.

## References

[bb1] Albrecht, M., Fiege, M. & Osetska, O. (2008). *Coord. Chem. Rev.* **252**, 812–824.

[bb2] Alexander, O. T., Duvenhage, M. M., Kroon, R. E., Brink, A. & Visser, H. G. (2021). *New J. Chem.* **45**, 2132–2140.

[bb3] Dolomanov, O. V., Bourhis, L. J., Gildea, R. J., Howard, J. A. K. & Puschmann, H. (2009). *J. Appl. Cryst.* **42**, 339–341.

[bb4] Farrugia, L. J. (2012). *J. Appl. Cryst.* **45**, 849–854.

[bb5] Feng, L., Wang, X. & Chen, Z. (2008). *Spectrochim. Acta A Mol. Biomol. Spectrosc.* **71**, 312–316. 10.1016/j.saa.2007.12.036 18289929

[bb6] Feng, L., Wang, X., Zhao, S. & Chen, Z. (2007). *Spectrochim. Acta A Mol. Biomol. Spectrosc.* **68**, 646–650. 10.1016/j.saa.2006.12.041 17395526

[bb7] Filik, H. & Apak, R. (1994). *Sep. Sci. Technol.* **29**, 2047–2066.

[bb8] Geffert, C., Kuschel, M. & Mazik, M. (2013). *J. Org. Chem.* **78**, 292–300. 10.1021/jo301966z 23270379

[bb9] Gloe, K., Stephan, H., Krüger, T., Möckel, A., Woller, N., Subklew, G., Schwuger, M. J., Neumann, R. & Weber, E. (1996). *Prog. Colloid Polym. Sci.* **101**, 145–148.

[bb10] Groom, C. R., Bruno, I. J., Lightfoot, M. P. & Ward, S. C. (2016). *Acta Cryst.* B**72**, 171–179. 10.1107/S2052520616003954 PMC4822653 27048719

[bb11] Kwak, S. W., Kim, M. B., Shin, H., Lee, J. H., Hwang, H., Ryu, J. Y., Lee, J., Kim, M., Chung, Y., Choe, J. C., Kim, Y., Lee, K. M. & Park, M. H. (2019). *Inorg. Chem.* **58**, 8056–8063. 10.1021/acs.inorgchem.9b00802 31120743

[bb12] Matsumura, M. & Akai, T. (1996). *Jpn. J. Appl. Phys.* **35**, 5357–5360.

[bb13] Mazik, M. & Geffert, C. (2011). *Org. Biomol. Chem.* **9**, 2319–2326. 10.1039/c0ob00960a 21321767

[bb14] Montes, V. A., Pohl, R., Shinar, J. & Anzenbacher, P. (2006). *Chem. Eur. J.* **12**, 4523–4535. 10.1002/chem.200501403 16619313

[bb15] Schulze, M., Löwe, R., Pollex, R. & Mazik, M. (2019). *Monatsh. Chem.* **150**, 983–990.

[bb16] Sheldrick, G. M. (2008). *Acta Cryst.* A**64**, 112–122. 10.1107/S0108767307043930 18156677

[bb17] Sheldrick, G. M. (2015*a*). *Acta Cryst.* A**71**, 3–8.

[bb18] Sheldrick, G. M. (2015*b*). *Acta Cryst.* C**71**, 3–8.

[bb19] Stoe & Cie (2002). *X-AREA*, *X-AREA Recipe*, *X-RED32* and *LANA*. Stoe & Cie, Darmstadt, Germany.

[bb20] Uhlemann, E., Mickler, W., Ludwig, E., Ludwig, E. & Klose, G. (1981). *J. Prakt. Chem.* **323**, 521–524.

[bb21] Uhlemann, E., Weber, W., Fischer, C. & Raab, M. (1984). *Anal. Chim. Acta*, **156**, 201–206.

[bb22] Yamada, H., Hayashi, H. & Yasui, T. (2006). *Anal. Sci.* **22**, 371–376. 10.2116/analsci.22.371 16733306

